# Supplementary material for: Male Wistar Rats Chronically Fed with a High-Fat Diet Develop Inflammatory and Ionic Transport Angiotensin-(3–4)-Sensitive Myocardial Lesions but Preserve Echocardiographic Parameters
Source: Int J Mol Sci. 2024 Nov 20;25(22):12474. doi: 10.3390/ijms252212474 (PMC11594684; doi:10.3390/ijms252212474)
Supplement: Supplementary file 1 [file ijms-25-12474-s001.zip › ijms-3129062-supplementary.pdf]

Supplementary material

Male Wistar rats chronically fed with a high-fat diet develop inflammatory and ionic transport Angiotensin-(3–4)-sensitive myocardial lesions but preserve echocardiographic parameters

Thuany Crisóstomo [1], Rafael Luzes [2], Matheus Leonardo Lima Gonçalves [3], Marco Antônio Estrela Pardal [4], Humberto Muzi-Filho [4], Glória Costa-Sarmento [4], Debora B. Mello [5] and Adalberto Vieyra [2,4,5,*]

[1] Leopoldo de Meis Institute of Medical Biochemistry, Federal University of Rio de Janeiro, 21941-902 Rio de Janeiro, Brazil; thuany.crisostomo@bioqmed.ufrj.br

[2] Graduate Program in Translational Biomedicine/BIOTRANS, Grande Rio University/UNIGRANRIO, 25071-202 Duque de Caxias, Brazil; rafael.luzes@abeugraduacao.com.br (R.L.); avieyra@biof.ufrj.br (A.V.)

[3] Grande Rio University/UNIGRANRIO, 25071-202 Duque de Caxias, Brazil; matheusunigran-rio@outlook.com (M.L.L.G)

[4] Carlos Chagas Filho Institute of Biophysics, Federal University of Rio de Janeiro, 21941-902 Rio de Janeiro, Brazil; marco.pardal20@gmail.com (M.A.E.P.); humbertomuzi@biof.ufrj.br (H.M-F.); sarmen-to@biof.ufrj.br (G.C-S.); avieyra@biof.ufrj.br (A.V.)

[5] National Center for Structural Biology and Bioimaging/CENABIO, Federal University of Rio de Janeiro, 21941-902 Rio de Janeiro, Brazil; debmello@cenabio.ufrj.br; (D.B.M.); avieyra@biof.ufrj.br (A.V.)

* Correspondence: avieyra@biof.ufrj.br

Content:

Figure S1. Quantification of GAPDH levels from immunoblotting assays.

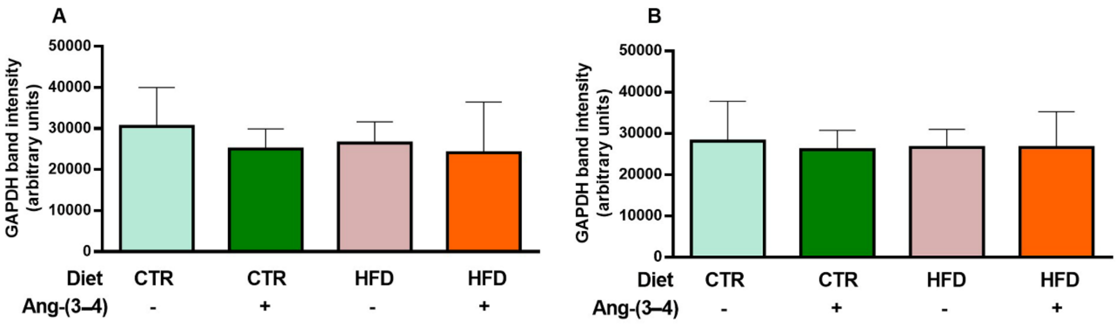

Figure S1. Quantification of GAPDH levels, loading control for proinflammatory cytokines IL-6 and TNF-α (Figure 6) and Ang II receptors (Figure 9) immunoblottings. (**A**) GAPDH levels used as loading control for IL-6 and TNF-α ($n = 6$). (**B**) GAPDH levels used as loading control for AT_1R and AT_2R ($n = 7–8$). Bars show the mean ± SD of the different preparations of cardiac microsomes. Differences were assessed using two-way ANOVA followed by Bonferroni's test. In **A**, *P* values are: 0.5575 (CTR vs CTR + Ang-(3– 4)), 0.6876 (CTR vs HFD), > 0.9999 (other comparisons). In **B**, all *P* values are > 0.9999.
